# Development of a plain-language guide for discussing breast cancer genetic counseling and testing with patients with limited health literacy

**DOI:** 10.1007/s00520-020-05800-7

**Published:** 2020-10-01

**Authors:** J. A. M van der Giessen, M. G. E. M. Ausems, E. van Riel, A. de Jong, M. P. Fransen, S. van Dulmen

**Affiliations:** 1 Division Laboratories, Pharmacy and Biomedical Genetics, Department of Genetics, University Medical Center Utrecht, Utrecht, The Netherlands; 2 Reading & Writing Foundation, Den Haag, The Netherlands; 3 Department of Public and Occupational Health, Amsterdam Public Health Research Institute, Amsterdam UMC, University of Amsterdam, Amsterdam, The Netherlands; 4 Research Institute for Health Sciences, Department of Primary and Community Care, Radboud University Medical Center, Nijmegen, The Netherlands; 5 Nivel (Netherlands Institute for Health Services Research), Utrecht, The Netherlands; 6 Faculty of Health and Social Sciences, University of South-Eastern Norway, Drammen, Norway

**Keywords:** Genetic counseling and testing, Plain language, Health literacy, Genetic literacy, Access to care

## Abstract

**Purpose:**

Due to limited health literacy and resulting ineffective communication between healthcare professionals and patients, not all eligible patients are offered breast cancer genetic counseling and testing. We aimed to develop a plain-language guide to increase effective communication about genetic counseling and testing with breast cancer patients with limited health literacy.

**Methods:**

Together with oncological healthcare professionals, we drafted a list of jargon words frequently used during (breast) cancer genetic counseling. In a focus group interview with breast cancer counselees with limited health literacy, who had received genetic counseling before, we reformulated these words in plain language. Low-literate individuals, who are not familiar with breast cancer care or genetic counseling, reflected on the draft of the guide. Completeness, acceptability, and perceived usability were tested in an online questionnaire among healthcare professionals.

**Results:**

The result is a plain-language guide for genetic counseling and testing with 33 frequently used jargon words and a reformulation of these words in plain language. Acceptability and perceived usefulness of the guide among healthcare professionals (*n* = 58) were high.

**Conclusion:**

The plain-language guide provides opportunities to facilitate communication about genetic counseling and testing with patients with limited health literacy and could enhance opportunities for patients to make informed decisions to participate in genetic testing. As the intention from healthcare professionals to use the plain-language guide is high, implementation of the guide in a real-life setting seems promising.

## Introduction

It is important that women at risk of carrying a mutation in a breast cancer gene are offered breast cancer genetic testing. It can help them to make decisions about their own treatment or prevention strategies and can have implications for their (healthy) family members, including future generations [1, 2].

Due to limited health literacy and resulting ineffective communication between healthcare professionals and patients, not all eligible patients are offered genetic counseling and testing [3–9]. Patients' limited health literacy and their lack of experience with the healthcare system were found to be barriers, making it difficult for patients to actively engage in taking healthcare decisions [10, 11] and is also associated with lower genomic related knowledge [12, 13]. Given that in the Netherlands over 36% of Dutch adults have low or limited health literacy [14], a sizeable proportion of patients lack adequate understanding of medical terms. Most health literacy projects have focused on patient factors, with relatively less emphasis on the communication skills of healthcare professionals [15, 16]. However, being able to correctly assess the patient’s level of health literacy is a prerequisite for effective communication. Research shows that there are significant gaps in knowledge, awareness, and skills to recognize limited health literacy among nurses and physicians [17–19].

In daily practice, jargon is overused in communication with patients and is a barrier to effective medical communication, especially when health literacy is limited or the topic is complicated [20, 21]. Avoiding jargon and using plain language seem promising strategies for effectively communicating health information.[22–24]. In the context of genetic counseling, it was found that the greater the use of technical terms, the greater the literacy demand of a genetic counseling session [25]. Guidelines or tools for the use of plain language may be a useful addition to medical consultations [26]. Although there are a number of plain-language word replacement resources, like a plain-language medical thesaurus [27], these tools are not sufficiently tailored to the context of (cancer-)genetics. In the context of genetics, the development of the REALM-G recognizes the need to identify which patients may be in need of communication in plain language because of limited health literacy [28]. However, it cannot be used as a tool to facilitate healthcare professionals to communicate effectively about breast cancer genetic counseling.

The specifics of plain language tools depend on the needs of patients, so it is critical to involve them in the development process [22]. But also involving healthcare professionals as intended end-users is crucial for effective implementation. Solutions designed in this way are more likely to be acceptable to both providers and end-users [29].

We aimed to develop together with breast cancer patients with limited health literacy and low-literate individuals a plain-language guide for healthcare professionals to effectively discuss breast cancer genetic counseling and testing. The following research questions were addressed:

1. What are plain language synonyms for jargon words frequently used in breast cancer genetic counseling and testing according to breast cancer patients with limited health literacy and low-literate individuals?
2. How do intended end-users (healthcare professionals) perceive the completeness, acceptability, and usefulness of a plain-language guide for genetic counseling?

## Methods

The development of the plain-language guide is part of the Erfo4all project, a project that aims to achieve equal access to breast cancer genetic counseling for all eligible patients. Within this project, we developed a blended training program for healthcare professionals, consisting of the following two successive parts: an online module and a group training [30]. In the group training, the teach-back method—a methodology used by healthcare professionals to check whether a patient understands what has been discussed—was used as a technique to identify patients with limited health literacy [31].

### Participants

The plain-language guide was developed step by step, using an iterative two-stage design. Breast cancer patients with limited health literacy, low-literate individuals, and intended end-users (breast surgeons, clinical geneticists, and specialized nurses) were actively involved.

### Instrumentation and procedures

#### Phase 1: focus group interviews breast cancer patients with limited health literacy and low-literate individuals

Together with breast cancer surgeons and specialized nurses (*n* = 59) who completed the Erfo4all training program [30] and a clinical geneticist and a genetic counselor from the Genetics Department of the University Medical Center Utrecht, we drew up a list of jargon words that are frequently used verbally and in writing during breast cancer genetic counseling consultations.

Subsequently, we conducted a focus group interview with breast cancer patients with a lower educational background or a limited level of health literacy and a personal experience with breast cancer genetic counseling and reformulated these words in plain language. Input from the focus group interview was used to develop a draft of the plain-language guide.

In a second group interview with low-literate individuals with no personal experience in breast cancer genetic counseling, the first draft of the guide was evaluated. We conducted this second group interview because there is evidence that a sizeable proportion of laypersons lack adequate understanding of several common terms used in medical consultations, do not understand phrases often used in cancer consultations, and cannot be assumed to have basic medical knowledge [32, 33]. Participants were asked to provide feedback relating to the comprehensibility of the preliminary version of the plain language guide thereby supported by an information letter in which the setting of breast cancer genetic counseling was outlined. Based on the feedback of low literate individuals, we refined the guide.

#### Participant selection

For the first focus group interview, we wanted to include patients with limited health literacy to provide input for a language guide adapted to their needs and abilities. Breast cancer patients who completed breast cancer genetic counseling at the Genetics Department of the University Medical Center Utrecht between March 2017 and October 2018 were invited.

Selection of these patients was done using background data that were registered on a checklist of the Erfo4all project. We selected patients that either scored low on health literacy or had a low educational attainment and migrant background, because these variables are known to be associated with health literacy competences [34]. Health literacy was assessed by asking patients the following validated question: “How often do you need help reading letters or information from your doctor, hospital, or other health institutions?” [35]. Inclusion criteria were as follows: no medical or social restriction for participation and able to speak Dutch. Eligible patients (*n* = 64) received a letter in plain Dutch to inform them about the aim and the procedure of the focus group interview. Within two weeks, a researcher contacted them by phone to ask if they wanted to participate. For the second group interview, low-literate adult individuals with no personal experience with breast cancer genetic counseling, recruited from the Dutch Reading & Writing Foundation, were invited. They also received a letter in plain Dutch to inform them about the aim and the procedure of the group interview and an invitation to the meeting. Ethical approval for the study was waived, but in line with the Declaration of Helsinki [36]. Furthermore, we asked the participants from both group interviews to sign a consent form, certifying that the information given is confidential, that participants understood the study information, and that they are aware of the fact that they can withdraw from the focus group interview at any time. They also gave permission to audio-record the interview.

#### Data collection during focus group interviews

Patients were asked to reflect on jargon words used during routine breast cancer genetic counseling. We asked which words were unknown or difficult, which words they recognized, and what they thought the meaning of these words was. Together with the patients, we rephrased difficult words concerning genetic counseling and testing in plain language until the participants were satisfied with the final formulation. During the interviews, we used the teach-back method as a strategy to ensure words and explanations are understood [37, 38]. In the group interview with participants from the Dutch Reading & Writing Foundation, the guide was discussed and tested on laypersons’ understanding. Both group interviews lasted 1.5 h and were audio-recorded, so they could be listened to independently by two authors (JG and SvD) to ensure no information was missed.

#### Phase 2: survey among intended end-users

In this phase, we aimed to explore intended end-users’ (breast cancer surgeons, specialized nurses, clinical geneticists, genetic counselors) perceptions of the plain-language guide on completeness, acceptability, and usefulness. The plain-language guide and a digital questionnaire were sent to 59 healthcare professionals involved in breast cancer care in three regions in the Netherlands, who participated in the Erfo4all training program, and to clinical geneticists and genetic counselors from the genetics departments in four academic centers in the Netherlands (*n* = 47). A cover letter informed them about the aim of the study and the importance of their input. We asked healthcare professionals if they noticed any unnecessary or missing words on the preliminary list. Furthermore, we asked about their acceptance of the reformulation in plain language, the perceived usefulness of the guide, and finally their intention to use the guide in daily practice. We used an adapted version of the USE questionnaire [39] to assess the acceptability and perceived usefulness, based on a 5-point Likert scale, ranging from (1) totally disagree to (5) totally agree. Open-ended questions were used to ask about their intentions to use the plain-language guide and to ask for suggestions to refine the guide on content or design.

#### Statistical analyses

All data from the questionnaires were entered in SPSS version 24.0. Categorical data, number of healthcare professionals, sex, and discipline are presented in numbers and percentages. Descriptive statistics were used to present outcome measures from the questionnaires.

## Results

### Outcomes phase 1: feedback from breast cancer patients with limited health literacy and low-literate individuals

#### Response

Of the 64 patients who were invited, 11 patients and four of their partners participated in the focus group interview. Table 1 shows the background characteristics of participating patients.

**Table 1.** Background characteristics of patients participating in the focus group interview

Sex	Male Female	2 9
Breast cancer	Yes No	7 4
Eligibility for genetic testing	Diagnostic DNA testing Predictive testing Did not meet criteria for testing	7 3 1
Educational level	Low Intermediate-1 Intermediate-2 High	4 7 0 0
Level of health literacy	Low Intermediate High	10 1 0
Migrant background	Yes No	2 9

All patients had a lower level of education (i.e., less than primary education, primary or lower secondary education) or were identified to have limited health literacy. Patients who did not participate explained that this was due to practical considerations, mostly involving their work schedule or transportation to the hospital. In the second session with participants from the Reading & Writing Foundation, three low literate individuals participated; one male and two females.

#### Reflection on jargon words and reformulation by (breast cancer) patients with limited health literacy (focus group interview 1)

Patients with limited health literacy stated that terms related to genetic testing are difficult to understand and sometimes ambiguous (e.g., “hereditary or genetic predisposition, what’s the difference?”). Moreover, the difference between a gene and DNA needed clarification. Jargon words “(gene) mutation” and “gene panel” are considered the most difficult and abstract words. A gene panel is associated with a group of individuals and not with a test that analyzes multiple genes at once for cancer-associated mutations (“I think we are in a gene panel right now”). According to the patients, it is important to be as specific as possible and to avoid abbreviations.

#### Reflection by low-literate individuals on the draft version of the plain-language guide (focus group interview 2)

Low-literate individuals considered most of the jargon words in the plain-language guide and in the patient information letter to be difficult (“these are all difficult words”). They stressed the importance of meeting the needs of patients with lay knowledge (“it’s another world, we have no idea”) and stated that most of the rephrased words on the plain-language guide are acceptable and understandable. Based on the patient information letter, participants from this group interview suggested four more jargon words and the reformulation of these words to add to the plain-language guide. Table 2 shows the primary list of jargon words, the reflection and reformulation by patients with limited health literacy, and the reflection on the draft of the guide by low-literate individuals.

**Table 2.** Reflection by breast cancer patients with limited health literacy and low-literate individuals in phase 1

Jargon words (frequently used)	Focus group 1 (*n* = 11) *patients with limited health literacy * *(reformulation of jargon words)*	Focus group 2 (*n* = 3) *low-literate individuals* *(reflection on reformulation)*	Focus group 2 *low-literate individuals* *(suggestion for extra jargon words to add to the guide)*	
BRCA 1	Name of one of the breast cancer genes. The abbreviation is from Breast-Cancer. A mistake in this gene causes an increased risk of breast cancer and ovarian cancer	Important to explain the difference between BRCA 1 and BRCA 2 and the connection between the illness and change in a gene	BRCA 1	
BRCA 2	Name of one of the breast cancer genes. The abbreviation is from Breast-Cancer. A mistake in this gene causes an increased risk of breast cancer and ovarian cancer (risk of ovarian cancer is lower than with a BRCA 1 mutation)		BRCA 2	
Cells	“Building blocks” of your body	Unclear that DNA is in the cells	Cells	
Clinical geneticist	Physician with a specialization in heredity	b	Clinical geneticist	
			To diagnose^a^	To determine if someone has a disease (e.g. breast cancer)
DNA	This contains all your personal characteristics. It is your blueprint or the recipe of your body	Important to explain abbreviations. DNA is associated with police work. Blueprint is a clear description.	DNA	
DNA test	A test to find out if there are any changes in your DNA	b	DNA test	
Familial breast cancer	When breast cancer is common in the family	b	Familial breast cancer	
Family history	The diseases that are in the family	b	Family history	
Family tree	A drawing of your family and relatives; father, mother, brothers, sisters, grandparents, and so on	b	Family tree	
Gene	A small piece of your DNA with a special characteristic, like the color of your eyes	b	Gene	
Genetic counselor	Someone who gives information and advice about heredity and genetic testing	The difference between geneticist and counselor is difficult. Do not use foreign words.	Genetic counselor	
Gene mutation	Change or mistake in a gene, in a piece of DNA	Most difficult word	Gene mutation	
Gene panel	A group of genes investigated at the same time	Panel is associated with a group of individuals	Gene panel	
Genetic predisposition	If a certain disease is in your family and you can pass it on to the next generation	b	Genetic predisposition	
Genetic test	Heredity test	Addition: DNA test	Genetic test	
Hereditary	Something your parents pass on to you; it is “in the family”	b	Hereditary	
Hereditary screening	Testing to find out if a certain disease is in your family	b	Hereditary screening	
Inheritance	How the disease is passed on within the family	b	Inheritance	
Increased risk	You are more likely to get the disease	Increased risk is just “bad luck”	Increased risk	
Mamma care	Breast care in the hospital	Explain the term “mamma.” It is associated with being pregnant, breastfeeding	Mamma care	
Mamma surgeon	A surgeon who is specialized in breast-care and cancer	b	Mamma surgeon	
Mutation	A change or a mistake	Most difficult to understand	Mutation	
Mutation carrier	Someone with a change or a mistake in one of the genes	It is helpful to use this word in a sentence.	Mutation carrier	
			Pathologic examination^a^	Examination of tissue and cells in a laboratory
			Risk factor^a^	Something that increases the chance of getting a disease
Screening	Medical exam to find out if there is an abnormality	Screening is associated with preventive	Screening	
Transmissible	Something in the family that can be passed on to the next generation, such as a disease, or eye color	b	Transmissible	
			Tumor^a^	Benign of malignant (cancer) growth

^a^Missing word added by target group of patients based on the information letter

^b^Clear description

### Outcomes phase 2: intended end-users’ feedback

Of the 106 healthcare professionals invited to participate, 66 responded (62%) of whom 58 completed the entire online questionnaire (55% of those invited). Table 3 shows the background characteristics of healthcare professionals who responded to the questionnaire.

**Table 3.** Background characteristics of healthcare professionals who responded to the questionnaire.

*n* = 66		*n*	%
Sex	Male	7	10.6
	Female	59	89.4
Discipline	Breast surgeon	5	7.6
	Specialized nurse	24	36.4
	Physician assistant	4	6.0
	Clinical geneticist	15	22.7
	Genetic counselor	7	10.6
	Other	11	16.7

Almost 17% of the healthcare professionals indicated that certain words on the preliminary list were unnecessary and almost 27% of them said that specific words in relation to breast cancer genetic counseling were missing. Their reflections were based on daily practice during breast cancer genetic counseling. Healthcare professionals also evaluated the plain-language guide on completeness, usefulness, and acceptance. They considered six words in the guide to be unnecessary, and they suggested that 11 words be added to the guide. According to the healthcare professionals, the following words were unnecessary: *familial breast cancer*, *genetic test*, *genetic counselor*, *gene panel*, *family tree*, and *mamma surgeon*. They suggested that the following words be added: *autosomal dominant inheritance*, *HER 2 positive*, *mammography*, *MRI*, *physician assistant*, *preventive examination*, *specialized nurse*, *receptor*, *sentinel lymph node*, *triple negative tumor*, *other breast cancer genes* (like *CHEK2*, *PALPB2*, and *ATM*). Based on daily practice and experience during the Erfo4all training sessions, the project team decided how to adapt the guide, in accordance with these suggestions.

More than half (57%) of the healthcare professionals stated that they had the intention to use the plain-language guide predominantly in consultations with patients with limited health literacy or a migrant background. Almost 65% of the healthcare professionals stated that they would share the plain-language guide with colleagues. Suggestions for adaption of the guide mostly concerned content and design, for example digitalizing the guide or to providing it in a pocket-sized format. Table 4 shows the perceived usefulness of the plain-language guide.

**Table 4.** Perceived usefulness of the plain-language guide for breast cancer genetic counseling and testing (GenGuide) of healthcare professionals who completed the questionnaire

*n* = 58	GenGuide facilitates the start of a conversation about GCT (%)	GenGuide will benefit patients (%)	GenGuide seems effective for discussing GCT (%)	GenGuide seems a useful addition to my work (%)	GenGuide seems time saving (%)	GenGuide seems easy to use (%)	Intention to use the GenGuide frequently (%)
Totally agree	13.8	31.0	13.8	13.8	3.4	12.1	6.9
Agree	43.1	58.6	44.8	55.2	27.6	62.1	50.0
Neutral	8.6	8.6	22.4	13.8	20.7	12.1	19.0
Not agree	24.1	1.7	12.1	8.6	36.2	6.9	17.2
Totally disagree	10.3	–	6.9	8.6	12.1	6.9	6.9

### Plain-language guide for breast cancer genetic counseling and testing

The result of the input from patients, low-literate individuals, and intended end-users is a plain-language guide for healthcare professionals (clinical geneticists, genetic counselors, and breast surgeons) with 33 jargon words reformulated in a clear and concise description in plain language (Table 5).

**Table 5.** Final version of the plain-language guide for breast cancer genetic counseling and testing

Jargon word	Plain language
BRCA 1	Name of one of the breast cancer genes. The abbreviation is from Breast-Cancer. A mistake in this gene causes an increased risk of breast cancer and ovarian cancer
BRCA 2	Name of one of the breast cancer genes. The abbreviation is from Breast-Cancer. A mistake in this gene causes an increased risk of breast cancer and ovarian cancer (risk of ovarian cancer is lower than with a BRCA 1 mutation)
Cells	“Building blocks” of our body
CHEK 2	Name of one of the breast-cancer genes. A mistake in this gene causes an increased risk of breast cancer, but this risk is lower than with the BRCA 1 and BRCA 2 genes.
Clinical geneticist	Physician with a specialization in heredity
Diagnose	To determine if someone has a disease (e.g. breast cancer)
DNA	This contains all your personal characteristics. It is your blueprint or the recipe of your body
DNA test	A test to find out if there are any changes in your DNA
Familial breast cancer	When breast cancer is common in the family
Family history	The diseases that are in the family
Family tree	A drawing of your family and relatives; father, mother, brothers, sisters, grandparents, and so on
Gene	A small piece of your DNA with a special characteristic, like the color of your eyes
Genetic counselor	Someone who gives information and advice about heredity and genetic testing
Gene mutation	Change or mistake in a gene, in a piece of DNA
Gene panel	A group of genes investigated at the same time
Genetic predisposition	If a certain disease is in your family and you can pass it on to the next generation
Genetic test	Heredity test, DNA test
Hereditary	Something your parents pass on to you; it is “in the family”
Hereditary screening	Testing to find out if a certain disease is in your family
Increased risk	You are more likely to get the disease
Inheritance	How the disease is passed on within the family
Mamma care	Breast-care in the hospital
Mammography	X-ray of the breasts
Mutation	A change or a mistake
Mutation carrier	Someone with a change or a mistake in one of the genes
Pathologic examination	Examination of tissue and cells in a laboratory
Physician assistant	Healthcare professional who independently takes over medical tasks from the clinical geneticist
Preventive examination	A medical examination to see if there are indications of a disease, such as breast cancer
Risk factor	Something that increases the chance of getting a disease
Screening	Medical exam to find out if there is an abnormality
Transmissible	Something in the family that can be passed on to the next generation, such as a disease or your eye color
Triple negative tumor	A special type of breast cancer, the tumor has special characteristics
Tumor	Benign or malignant (cancer) growths

## Discussion

In this study, we developed a plain-language guide based on clinical practices and tailored to the needs and preferences of patients with limited health literacy and low-literate individuals. Based on their input and preferences, an elaborate list of jargon words was reformulated in plain language. This is useful because when communicating with patients, healthcare professionals have a tendency to use medical jargon. Avoiding the use of medical jargon and instead using plain language can overcome important barriers in discussing breast cancer genetic counseling and testing. Such a guide might help healthcare professionals discuss (referral to) breast cancer genetic testing in a more comprehensible way. This is not only important for patients with limited health literacy or low literacy, but in communication with all patients. Especially because most healthcare professionals experience difficulties in recognizing limited health literacy[19].

Other studies have described the development of a plain language support tool for cancer clinical trials or plain language summaries of scientific articles [23, 40] and found that this could play an important role in the patient-physician dialogue. However, these studies were merely focused on patient empowerment and not directly on improving communication behavior from healthcare professionals. To our knowledge, this is the first plain-language guide in the context of genetics, developed with a focus on healthcare professionals’ behavior. Moreover, in the previous studies, reformulation in plain-language was not based on preferences and suggestions from patients with limited health literacy or low health literate individuals.

### Study limitations

Methodological considerations of our study mainly concern the selection of jargon words for the preliminary list. This selection was based on suggestions of healthcare professionals and not generated by listening to actual encounters with patients with limited health literacy. This may be a shortcoming of our study; however, the frequently used jargon words on the list were derived from the Erfo4all group training sessions together with breast surgeons and specialized nurses. Based on eight training sessions, these jargon words were considered to be representative. In the process of rephrasing jargon words, the focus was on the input from patients with limited health literacy and low-literate individuals. Healthcare professionals just reflected on the draft of the guide for practical implications and to increase the chance of a successful implementation. The intention of healthcare professionals to use the guide was relatively low (57%). Furthermore, we did not ask healthcare professionals to explain their answer in an open-ended question, so a valid explanation for the low intention to use rate is unclear, which is a shortcoming of our study. However, the perceived usefulness of the guide was high, so we are confident that more healthcare professionals will actually use the guide after implementation in daily practice.

The group of healthcare professionals that completed the questionnaire consisted mostly of clinical geneticists and specialized nurses. As breast surgeons were underrepresented in this study, the results on the usefulness and acceptability of the guide may not be entirely representative for this group. However, the feedback from specialized nurses who closely work together with the surgeons can be considered as a reflection of the acceptability of the plain-language guide in routine cancer care.

### Practice implications

The use of plain language can improve communication with patients with limited health literacy and provides opportunities for these patients to make informed decisions to participate in genetic testing. Our plain-language guide could improve communication about genetic testing with patients with limited health literacy among a diverse group of healthcare professionals involved in breast cancer care. Surgeons and specialized nurses discuss referral to genetic counseling with eligible breast cancer patients and after referral clinical geneticists and genetic counselors discuss genetic testing and the possible consequences. As genetic testing becomes further integrated into oncology, surgeons and medical oncologists are increasingly discussing the options and possible outcomes of genetic testing with patients and request these tests themselves. This results in a growing need among healthcare professionals involved in breast cancer care to communicate genetics information and facilitate decision making in a short time frame [41]. Discussing the consequences of genetic testing with patients with limited health literacy is time-consuming. Our plain-language guide is expected to be helpful to discuss genetic counseling and testing with these groups of patients more effectively.

We believe that the process for development of a plain-language guide can be translated to other health care context, because most of the terminology used in healthcare can be confusing for patients, especially for patients with limited health literacy or at times of distress when people may struggle more than usual to take in information [42, 43]. For implementation in daily practice, we will take into account the suggestions from healthcare professionals to digitalize the guide and to provide the guide in a pocket-sized format.

### Research recommendations

It seems feasible to develop a plain-language guide based on frequently used jargon words in daily practice and reformulate these words based on preferences and understanding from patients with limited health literacy and low-literate individuals. Future research should focus on testing the plain-language guide in a real-world setting and on the effect on patient activation and making informed decisions about participating in cancer genetic counseling and testing. Although other studies suggest that health literacy affects decision making in healthcare, more research is needed on how the use of plain language and specifically how a plain-language guide for healthcare professionals may influence the decision-making process to participate in (breast) cancer genetic testing. It might be interesting to explore opportunities to make the plain-language guide available for patients.

Next to the use of jargon or technical terminology, also other language characteristics of the medical dialogue, such as general language complexity or dialogue pacing, density, and interactivity play a role in patients’ understanding about genetic information [25]. It is worthwhile to take these into consideration for future research. Finally, although the plain-language guide was well received by intended end-users, we have not yet assessed the actual use in daily practice. It would be interesting to find out if assessment of patients’ literacy level with the REALM-G [28] prior to medical consultation will contribute to the use of the plain-language guide.

## Conclusion

In this study, we described the development process of a plain-genetic language guide for breast cancer genetic counseling. Our study showed that reformulation of frequently used jargon words in breast cancer genetic counseling and testing, together with patients with limited health literacy and low-literate individuals, is feasible. The result is a plain-language guide for healthcare professionals to discuss breast cancer genetic counseling in words that are understandable for these groups of patients. The collaboration with breast cancer patients in the reformulating process provides valuable insights into plain language synonyms from patients’ perspective. Furthermore, lay views often differ from those of patients and healthcare professionals, so reflection on the plain-language guide by low-literate individuals with lay knowledge provided an extra check on the formulation and comprehensibility of the guide.

Reluctance on the part of healthcare professionals to use a new tool is a risk in implementation. In the development of the plain-language guide, intended end-users (specialized nurses, breast surgeons, clinical geneticists, and genetic counselors) were actively involved. They brought in frequently used words, evaluated the guide, reflected on a draft version, and rated the guide regarding its usefulness and acceptability. The plain-language guide appears to be acceptable and useful, so implementation in daily practice in genetics as well as in mainstream oncology services seems worthwhile and feasible. This is important, because patients are increasingly urged to become involved in decision making, like the decision to participate in genetic counseling and testing. Therefore, attention for health literacy deficits, by using plain language, by speaking in words easily understood by patients, is a necessary, first step.
